# Vulnerable learners in the age of COVID-19: A scoping review

**DOI:** 10.1007/s13384-020-00409-5

**Published:** 2020-11-27

**Authors:** Catherine F. Drane, Lynette Vernon, Sarah O’Shea

**Affiliations:** 1 National Centre for Student Equity in Higher Education, Curtin University, Kent Street, Bentley, WA 6102 Australia; 2 School of Education, Edith Cowan University, Mt Lawley, WA Australia

**Keywords:** COVID-19 pandemic, Education, Vulnerable young people, Disadvantage, Scoping review

## Abstract

This scoping review provides an overview of COVID-19 approaches to managing unanticipated school closures and available literature related to young people learning outside-of-school. A range of material has been drawn upon to highlight educational issues of this learning context, including psychosocial and emotional repercussions. Globally, while some countries opted for a mass school shut-down, many schools remained open for students from disadvantaged backgrounds. This partial closure not only enabled learning in smaller targeted groups but also offered a safe sanctuary for those who needed a regulated and secure environment. In Australia, if full school closures were to be enforced over a long period, a significant proportion of students from more vulnerable backgrounds would likely experience persistent disadvantage through a range of barriers: long-term educational disengagement, digital exclusion, poor technology management, and increased psychosocial challenges. This scoping review combines research on technology availability and learning, with analysis of the long-term educational impacts of navigating the COVID-19 disruption.

## Introduction

The coronavirus (COVID-19) pandemic has created huge worldwide repercussions, impacting economies, health sectors, and education systems, as noted by the United Nations Educational, Scientific and Cultural Organization (UNESCO 2020a,b,c). To address the significant disruption to learning and educational environments, UNESCO (2020b) swiftly developed ten key recommendations to ensure that learning across the globe remained relatively uninterrupted during the COVID-19 crisis. These recommendations span the whole learning sphere, including the well-being and educational needs of learners, as well as the emotional health of educators and the need for common directions/guidelines for educational institutions. Nationally, the Australian federal government responded to calls for mass school closures by commissioning research to inform the community of the effects for vulnerable young people.^1^ The way in which various countries and educational systems responded to this global pandemic provided the impetus for this scoping review with particular reference to how mass school closures could impact our most *vulnerable* learners. The responses to the pandemic have been evolving and fluid; however, what has become clear is that the COVID-19 pandemic has exacerbated the multiple and profound educational divisions that already exist globally (Beaunoyer et al. 2020; Chandra et al. 2020). This article will consider these divisions and also reflect upon strategies that were employed during school closures to counter the long-term and detrimental consequences arising from this health and economic crisis (Beaunoyer et al. 2020; Chandra et al. 2020).

In an attempt to contain the COVID-19 virus and reduce its spread globally, 191 countries instigated nationwide school closures (UNESCO 2020c), affecting approximately 91.3% of students, around 1.5 billion students worldwide. In response to these school closures, many countries adopted online modalities to ensure continuity in learning; however, this adjustment heralded growth in concerns over how students from socially and financially disadvantaged backgrounds would be educationally, emotionally, and socially impacted by online learning. Questions arose regarding the best ways to support vulnerable students to continue their education. This scoping review serves as an informed basis to understand the educational impacts of the disruption caused by COVID-19, specifically for young people who may be living in disadvantaged contexts within Australia. In a situation characterised by constant change, this scoping review provides a baseline of the literature and research moving forward, synthesising currently available information from a range of sources. This article commences a dialogue about how unforeseen and global crises might be managed moving forward. In saying that, the authors are mindful that drawing recommendations from a transnational organisation such as UNESCO can be problematic. Any policy borrowing or lending that results in an unproblematic application of policy to differing educational contexts needs to be challenged, as policy should always be informed by the 'local' rather than the global, drawing upon place-based knowledges rather than general assumptions (Portnoi 2016). However, at the time of writing this paper, the UNESCO report provided the most accessible macro-level analysis of how to address the educational challenges engendered by COVID-19.

This review marks a starting point from which informed conversations can evolve, a means to consider both what is already known about educational differences across student populations as well as the ways in which policy decisions to manage COVID-19 disruption influenced teaching and learning contexts. The review highlights the major implications of being educationally at risk in Australia and details the issues impacting our more vulnerable student populations within the existing national school system. It explores possible consequences for vulnerable students of a mass school closure across Australia. The strategies and approaches adopted by other countries to manage these issues will also be outlined, including emerging good practice in what became the first global mass school closure of its time.

In Australia, certain individuals or groups experience vulnerability; this varies according to personal context and depends on the circumstances surrounding the individual. Notably, the social and economic conditions in which people live, learn, work, and play are the social determinants of health and educational disadvantage (Reay 2017). According to research, the more individuals are exposed to different forms of disadvantage, both social and material, the poorer the health and developmental outcomes, especially related to education (Goldfeld et al. 2018). Young people who have been exposed to more disadvantage than their peers have been described in terms of vulnerability (Arora et al. 2015). Vulnerability can result from forms of stratification and further contextualised in terms of the unfulfilment of basic rights (Skinner et al. 2006). Notably, receiving an education is one of the fundamental human rights for all children (UNGA 1959). For this paper, the term *vulnerability* has been deliberately chosen over *disadvantage* to recognise the multiple external factors that can impact on the life course of an individual learner.

Vulnerability is a multidimensional construct that is "embedded in complex social relations and processes" (Hilhorst and Bankoff 2006, p. 5). Therefore, vulnerability positions individuals in relation to each other, within broader systems of social disadvantage. For example, social vulnerability refers to the resilience of communities when confronted by external stressors such as the complex and cascading effects from COVID-19 disruption. It involves varying levels of access to resources such as information, knowledge, and technology, in order to prepare for, cope with, and recover from external stressors. A large contributor to social vulnerability is social stratification, particularly evident in the Australian education system where students from more materially wealthy backgrounds tend to go to high fee-paying schools (Perry and McConney 2013). Conversely, low-fee-paying schools are often less well resourced and may have student populations that encounter varying levels of economic vulnerability with low or limited household incomes (Perry 2018). Such economic vulnerability can also have detrimental impacts on educational outcomes, leading to criticisms that broad-based testing, such as PISA, is not an educational measure but an economic one (Niyozov and Hughes 2019).

Being mindful of context is key to understanding the specific needs of communities that are materially disadvantaged. Factors such as overcrowding, poor health, difficulties with community safety, and unemployment may impact more significantly on learners within low socioeconomic status (SES) communities (Griggs et al. 2008; Pinoncely 2016). Indicators of vulnerability must then consider a diversity of issues including the difficulties associated with coping with change, being unfamiliar with the cultural or social capitals valued in mainstream educational settings or experiencing lower levels of community interconnection, trust, and resource sharing (Alwang et al. 2001; Larsen 2013). Such risk factors can entrench communities in poverty and social disadvantage (Vinson et al. 2015). As a result, vulnerable young people can be exposed to an increased range of social, emotional and behavioural issues (Edward and Baxter 2013) which negatively impact on the ways in which these learners navigate and engage with the learning environment, when compared to their higher SES peers (Edward and Baxter 2013). The challenges wrought by COVID-19 have only added to the already identified risks for vulnerable young people.

Importantly though, when considering social contexts and different communities, it is vital not to slip unintentionally, into discourses of deficit. As Oikonomidoy (2015) explains, if we focus only on 'existing macro-level categories' (p. 110) such as race, gender, or class, in explorations of human behaviour, then we run the risk of assuming a level of powerlessness or interdependence in individual actors. While vulnerable young people may be more at risk educationally, it is important not to problematise the individual or assume that this is collectively a group in need of assistance. Instead, theorists such as Yosso (2005) and Lareau (2011) identify how diverse forms of capitals and strengths both inform and underpin actions in different community settings. For example, Yosso refines and expands Bourdieuian notions of cultural capital to propose a strengths-based model (Community Cultural Wealth) that identifies forms of capital that are often unrecognised within the educational landscape, yet arguably provide rich foundations that students can build upon to enact success. Unfortunately, such cultural strengths may be undervalued in most formal educational settings, which creates perceptions of learners as deficient in requisite skills and needing to be 'filled up' with appropriate knowledges. A process that essentially ignores or undervalues the existing 'experiential capitals' (O'Shea 2018) held by individuals, regardless of background or wealth.

Research across the Australian educational sector has indicated that a "cycle of intergenerational disadvantage" can be seen repeating itself in the lives of many young people from low socioeconomic backgrounds (Mission Australia 2017, p. 1). Family characteristics of each student within individual schools are recorded on enrolment and contribute to the Index of Community Socio-Educational Advantage (ICSEA) for each school. Illustratively, the school ICSEA indicates how socially segregated Australian schooling has become (Kenway 2013; Perry 2018; Perry and McConney 2013). The ICSEA is calculated using Australian Bureau of Statistics (ABS) data and draws on education, occupation, income, ethnicity, and location of student household (Australian Curriculum, Assessment and Reporting Authority 2015). Nationally, the mean ICSEA score is 1000, and one standard deviation from the mean is equal to 100. Schools with extreme disadvantage are scored around 700 with elite schools scoring up to 1300. When learning outcomes are measured between schools above and below the mean ICSEA, a highly differentiated system emerges whereby students in schools situated in low socioeconomic areas experience barriers to access academic curriculum, learning resources (especially technology-related), and quality pedagogy to support and encourage high academic expectations (Lamb et al. 2001; Naylor and James 2015; Vernon et al. 2019). Educational attainment levels (measured through PISA and other national assessment schemes) and school completion rates are consistently lower in high schools situated in low socioeconomic areas, resulting in fewer students transitioning to university, and higher levels of youth unemployment (Hérault and Kalb 2009; Mission Australia 2017; Vernon et al. 2019). Similarly, this situation is also reported in other countries, including the UK (Reay 2016, 2017).

Data from the ABS show that as of 2019 there were 3,948,811 students enrolled in 9503 schools, with 2,263,207 primary students and 1,680,504 secondary students. Mass school closures thus have the potential to impact nearly four million students (ABS 2019; Drane et al. 2020). The potential effects are varied, contingent upon the social and economic capacities of schools themselves and their student characteristics. Certainly, the gap for students from more socially disadvantaged backgrounds may be exacerbated by the very speedy global mass movement to online and off-site education (Chandra et al. 2020). As schools (and universities) increasingly closed their physical locations, these differences in resources, and student characteristics, resulted in differences in the learning opportunities afforded to, or accessed by, young people, with increased risk for students in households experiencing economic disadvantage (Chandra et al. 2020). Within Australia, the lowest quintile for family income comprises 20% of the total student population, which equates to approximately 800,000 school students (ABS 2019). Therefore, a significant proportion of Australian students from more vulnerable backgrounds were impacted when mass school closures occurred at the height of the COVID-19 lockdown (Drane et al. 2020). For schools providing for students experiencing socio-educational disadvantage, the division of resources and capacity to cope with change is further exacerbated when considering situational characteristics, whether students are in government or non-government schools, metropolitan or country schools, as well as each state's education governance. As a case study, a closer examination of schools in the Department of Education-Western Australia (DOE-WA) revealed schools to be spatially diverse and very heterogeneous, with significant differences in context and socioeconomic status dependent upon the characteristics of individual regions (see Table 1; DOE-WA 2020). For example, the proportion of students in the lowest quintile in the northern metropolitan region is 1.6%. In contrast, for students in schools in the south metropolitan region, 6.9% of students are in the lowest quintile. Additionally, most of these students are in government schools with ICSEAs well below the average of 1000 (DOE-WA 2020).

**Table 1:** Demographic and socioeconomic analysis of Western Australian schools for semester 1—2020

	Primary number students	Primary %	Secondary number students	Secondary %	Total school students
WA school students semester 1 2020					
Total number (school *N* = 1135)	277,933	59.5	189,384	40.5	467,317
High SEIFA	135,248	28.9	100,619	21.5	235,867
Low SEIFA	142,685	30.5	88,765	19.0	231,450

	Primary number students	Primary %	Secondary number of students	Secondary %	Lowest quintile total students
Lowest quintile (SEIFA) WA school students semester 1 2020					
Number schools in lowest quintile = 186**/**total number schools = 1135	34,044	7.3	21,418	4.6	55,462
Government schools (*n* = 137/805)^a^	24,981	5.3	13,310	2.8	38,291
Non-government schools (*n* = 49/312)^a^	9063	1.9	8108	1.7	17,171
North metropolitan (*n* = 21/360)	5600		1915		7515
South metropolitan (*n* = 68/373)	18,728		13,523		32,251
South west (*n* = 22/139)	2783		2325		5108
Wheat belt (*n* = 21/76)	2568		1981		4549
Midwest (*n* = 7/60)	576		274		850
Goldfields (*n* = 16/48)	1459		428		1887
Kimberley (*n* = 26/44)	1751		860		2611
Pilbara (*n* = 5/35)	579		112		691
Metropolitan (*n* = 89/733)	24,328	5.2	15,438	3.3	39,766
Country (*n* = 97/402)	9716	2.1	5980	1.3	15,696

*SEIFA* socioeconomic indexes for areas

^a^*n* = 18 community kindergartens (0 number in lowest quintile)

Although this case study highlights the Western Australian context, other states and countries have significant differences between regions with clusters containing high proportions of students from low SES backgrounds (Chandra et al. 2020; Perry 2018). As one school principal summed up after just three weeks of school closure in Irish schools, the potential impacts on vulnerable school students were both diverse and profound:

Our school is in an area of severe disadvantage. Many of our children's homes would suffer from food poverty. The children come to school hungry. Many attend the after-school project where they get their dinner, this too has closed. Never mind the effect on their education these children could be starving and spending more time in homes with addiction and violence. School is their safe place. (Burke and Dempsey 2020, p. 42)

Undoubtedly, those students already experiencing economic or other forms of vulnerability must be carefully considered for any future school closure within Australia. Fortunately, Australian educators were in a position to consider *common learnings* derived from countries that had school closures for extended periods and had already introduced a united national shift to learning in the home (e.g. Burke and Dempsey 2020). Many different factors impact on the transition to remote or off-site learning for all learners, especially given the existing contexts and differences noted for students from more vulnerable backgrounds, with the risk of broad educational disadvantage being particularly influential. The risks and barriers for students who could not attend school due to the pandemic are considered in the next section, along with details of how countries addressed the impacts of school closure to mitigate the contexts and complexities of students' environments.

### Risk of long-term educational disengagement

Central to effective learning, student success, and well-being is student engagement, including behavioural, emotional and cognitive engagement (Fredericks et al. 2004). Within the school community, the teacher–student relationship most often forms the basis for student engagement (Wang and Eccles 2012). As face-to-face teaching fosters (or impedes) the teacher–student relationship, all three components of student engagement, namely behavioural, emotional and cognitive, are impacted upon during mass school closures. Positive face-to-face interactions with teachers and peers at school increases the subjective valuing of students' learning (Wang and Eccles 2012). This is why immediate and constructive teacher feedback in an online environment is vital to stabilise a student's engagement with their education. This necessary feedback may be absent for those learners who have limited or no access to computers, or the Internet, and are isolated in their homes, restricting their ability to connect online and "see" their teachers (Chandra et al. 2020). Therefore, disasters (such as COVID-19), which displace children from their schools, will likely impede the teacher's ability to provide the requisite social support needed for all their students (Motti-Stefanidi 2019).

Teacher social support includes interactions that convey appreciation, respect, and caring within the teacher–student interactions leading to engagement (Wang and Holcombe 2010). When students work collaboratively in the school environment with a well-developed sense of their student voice, they can expand their knowledge base through positive cognitive and emotional interactions (Cunninghame et al. 2020; Järvelä et al. 2016). Again, for students learning in an isolated environment, and unable to access video conferencing resources, the absence of these positive interactions and feedback from their school community may influence both their engagement and emotional well-being. The extent to which schools maintained positive teacher–student relationships despite the fluctuating danger from COVID-19 impacted upon student engagement within schools. To ensure more students could get the most out of their education, many schools adapted to the challenges of COVID-19 by creatively engaging with their school communities to maintain important ceremonies during the pandemic (Masten and Motti-Stefanidi 2020). For many students, engagement includes developing the desire for further study and the expectation among students to transition from high school to higher education (Cunninghame et al. 2020).

Disengaged students can experience adverse academic and social outcomes, such as lower achievement and disruptive behaviour (Simpkins et al. 2015). Students from disadvantaged backgrounds are reported as being more likely to experience markers of disengagement, such as daily absence, disruptive behaviour, and poor school connectedness (Hancock and Zubrick 2015). School connection is a protective factor for many students and is associated with a reduction in risk-taking behaviours, as well as increases in school attendance and academic achievement (Simpkins et al. 2015). Many young people in more vulnerable contexts already have a precarious relationship with education (Harwood et al. 2017), so there was a strong possibility that these cohorts may further disengage from learning if the curriculum content was only provided online (Burke and Dempsey 2020). In summary, a loss of school connectedness, due to school closures, may exacerbate the risk of educational disengagement, especially for vulnerable young people. This is compounded for those children in care, or those moving between households or locations, as often school is the only constant in their lives. Without the presence of routine or essential pastoral *care*—due to school closures—these young people may permanently disengage from learning (Baker 2020).

One response to this risk of educational disengagement is to sustain meaningful communication between schools and families. One recent report on the social and relational impacts of national school closures in Ireland, based upon survey responses derived from over 2800 school leaders and principals, found that a number of school leaders identified how proactively seeking feedback from parents concerning the educational and emotional needs of the student within the family was one crucial way to sustain connection between schools and their communities (Burke and Dempsey 2020). In Ireland, many schools outlined the need for a more collective approach to learning, including involving family members in the co-design of learning tasks and activities. This echoes the *students as partners* approach employed within the Australian university sector (Matthews 2016; O'Shea et al. 2020), to work productively with parents and children to design and develop curriculum that is manageable within the home environment and responsive to the learner's needs. The *students as partners* approach identifies the expertise or cultural strengths of the learners themselves and focusses on learning as collaboration both within and outside the classroom. This is a relational approach to learning ascertained by a *joint ownership* of the learning process, negotiating the act of teaching as *doing with* rather than *doing to*. In the case of schools, involving parents in this process allows a more comprehensive and holistic partnership to evolve.

To facilitate this co-operation, the Spanish educational system made several communication platforms and apps available (e.g. Edugestio) which enabled all parties (teachers, parents/caregivers, and students) to co-create the learning process (UNESCO 2020d). Such initiatives echo two of UNESCO's ten recommendations to ensure learning remains uninterrupted during the pandemic (UNESCO 2020b, p. 1), namely "prioritise solutions to address psychosocial challenges before teaching; create communities and enhance connection". In the face of continued uncertainty, strengthening the partnerships which were created during the COVID-19 school disruptions and leveraging the capacities within the communities will go a long way to ensuring increased flexibility and adaptability in our schools, so they are ready for future unanticipated changes or disruptions.

## Emotional well-being and anxiety

As schools made the shift to online learning, supporting students' social and emotional well-being became imperative (Brown et al. 2020). Although some students were faced with online learning issues attributed to technology use, there were also emotional challenges associated with change, including a shift to off-site learning. The emotional consequences related to school closures cannot be underestimated. Anxiety disorders are the most common mental, emotional and behavioural problems among young Australians (Australian Institute of Health and Welfare 2016). Specifically, 13.9% of children and adolescents experience a mental health disorder, including 6.9% experiencing anxiety (Australian Institute of Health and Welfare 2016). As students lost school connectedness due to being physically distanced from school, or having to maintain a social distance from teachers and peers, there was a sense that adults and/or peers in their school were no longer concerned about them as an individual or concerned about their learning. As such, psychological distress such as anxiety and depression has increased during the COVID-19 disruption (Holmes et al. 2020; Pikulski et al. 2020).

Further implications of learning off-site relate to children's emotional safety, as schools may provide a safe and nurturing haven for many students; physical and social isolation may deny them this emotional refuge. Many parents have also experienced psychological distress from personal disruptions due to COVID-19, such as unemployment and financial strain, and for some parents this may also be coupled with ineffective coping mechanisms, further exacerbating psychological distress (Caplan and Schooler 2007; Puterman et al. 2009). Adding to the burden of this fraught situation is an expectation that parents assume the role of educator within the household, a role that many parents may not be equipped for physically or emotionally (Burke and Dempsey 2020; UNESCO 2020b). Proactive emotional support for the families most impacted by this situation involves managing emotional, financial and logistical challenges in a multisystem approach across the community to support vulnerable families (Masten and Motti-Stefanidi 2020). Children already impacted by poverty and facing the challenge of navigating a pandemic required communities to step up to supply support for families and this has been demonstrated across Australia (e.g. Western Australian police, prisoners, and local businesses supplied furniture and computers to Mt Barker Community College students so they could study at home; Makse 2020). The multisystem mobilisation that occurred between schools and their communities during the COVID-19 pandemic was able to support vulnerable families. Leveraging these systems and processes in the future will build resilience and capacity to cope with future disruption (Chandra et al. 2020).

UNESCO (2020b, p. 1) highlights the importance of addressing the psychosocial challenges associated with the pandemic and recommends that this take priority over teaching, describing the necessity to "ensure regular human interactions, enable social caring measures, and address possible psychosocial challenges that students may face when they are isolated". Strategies could include utilising existing student mentoring programs already established across the Australian university sector. For example, the Australian Indigenous Mentoring Experience (AIME) program has successfully mentored young people in both primary and high school settings for over a decade (O'Shea et al. 2016); and e-mentoring programs, established in many universities, have supported prospective or commencing students (Jardine et al. 2016). Through existing university and community mentors, additional support and advice may be offered to school students online or via telephone. Another strategy may involve undergraduate (student) teachers who may have their intern practicums on hold due to COVID-19, and who might assist schools in an online mentoring capacity, with reciprocal benefits possible for both school students and university undergraduate teachers, as suggested by Sonnemann and Goss (2020).

## Digital inclusion

Digital inclusion is based on the premise that all individuals and communities, including those most disadvantaged, have access to, and use of, communication technologies (Thomas et al. 2019). A lack of digital skills and digital access can have a negative impact on learning (Chandra et al. 2020). As evident in the Australian Digital Inclusion Index (Thomas et al. 2019) which measures digital inclusion in three discrete ways (access, affordability, and digital ability), a digital divide exists between students from low and high socio-educational backgrounds. Notably, inadequate technology access negatively impacts students with different levels of access to financial resources (Thomas et al. 2019). This index indicates gradual growth across the three dimensions in Australia; however, digital inclusion remains consistently low in households of lower income (Thomas et al. 2019).

**Fig. 1:**
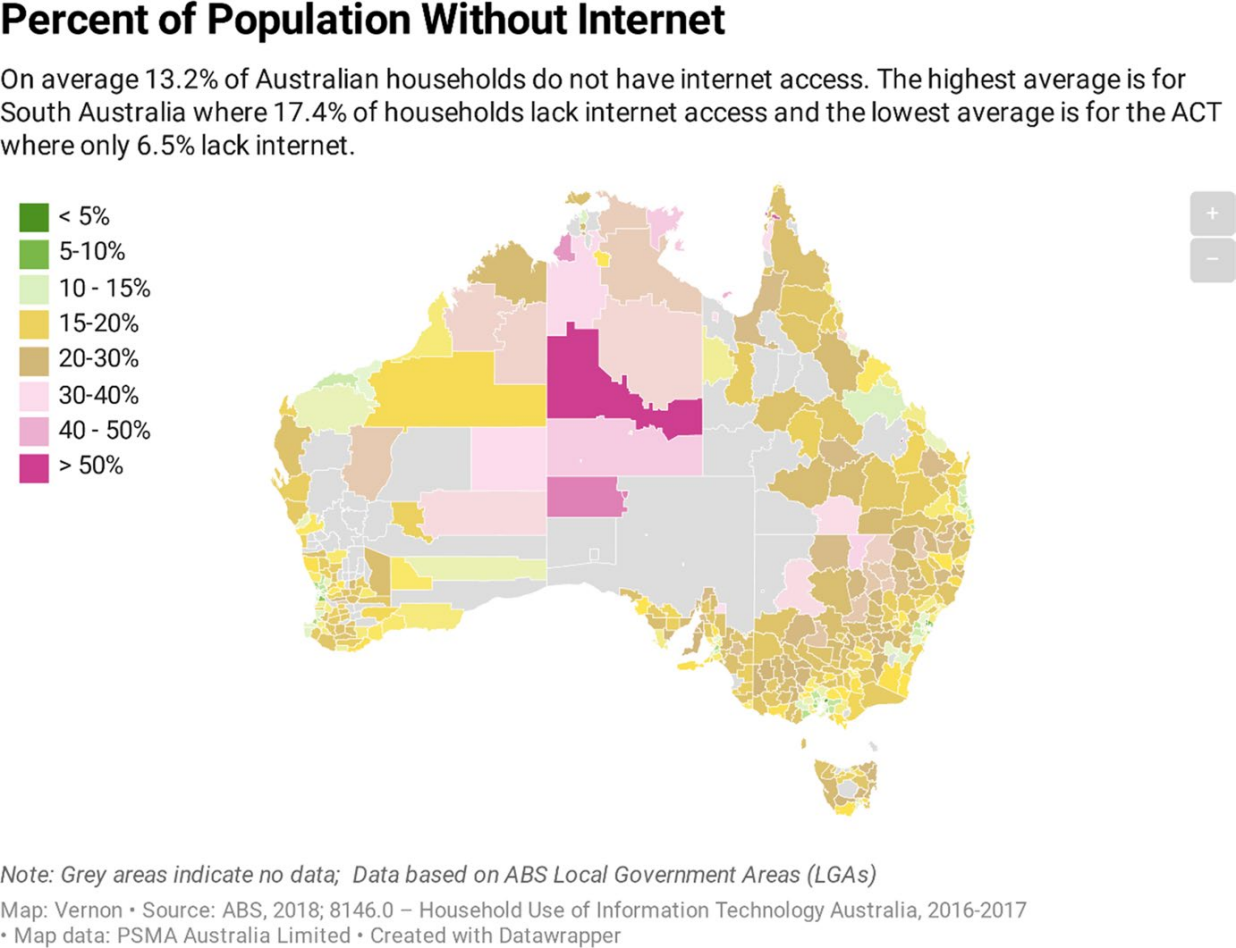
Population without Internet access (ABS 2018)//datawrapper.dwcdn.net/CHx4K/3/

**Fig. 2:**
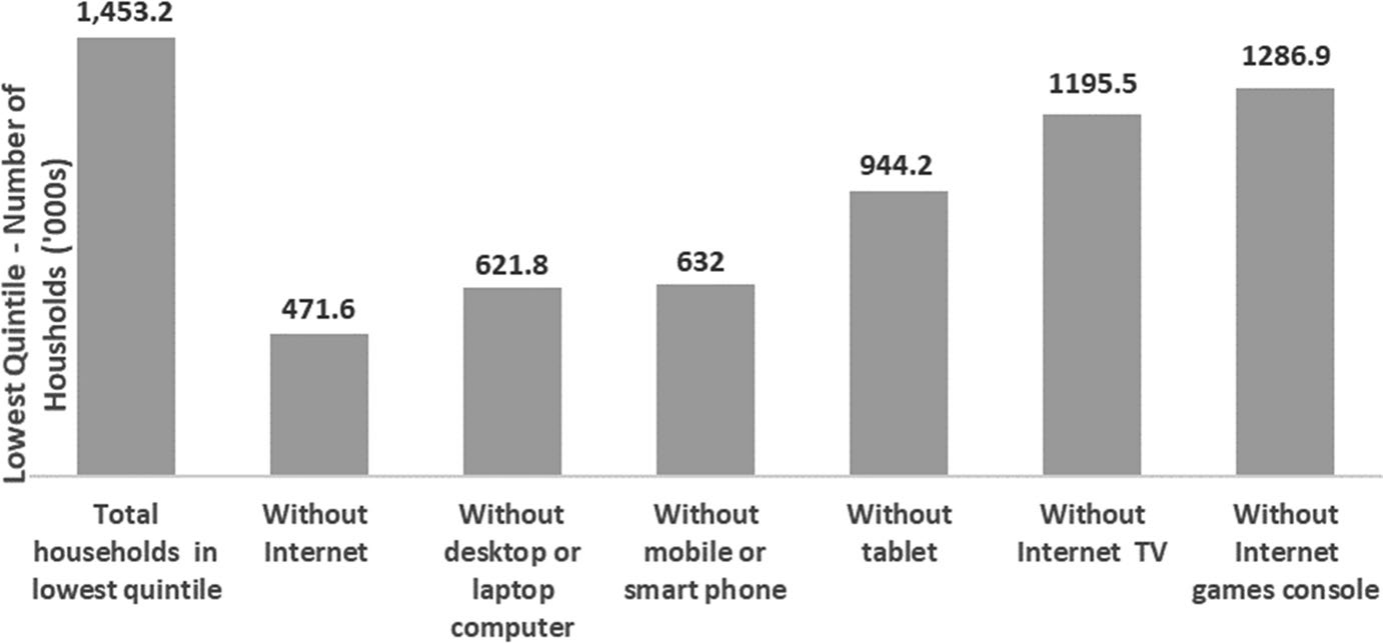
Information Technology access for Lowest Quintile for household income (ABS 2018)

According to the ABS (2018), on average, 13.2% of Australian households do not have access to the Internet. More than 90% of households in advantaged areas have an Internet connection, and less than 40% of households in disadvantaged geographical areas are connected (See Fig. 1; Drane et al. 2020). Approximately 471,600 households from the lowest quintile of household income (i.e. the lowest 20% of the population) have no access to the Internet, and approximately 621,800 households in the lowest quintile do not have access to a laptop or desktop computer. Insufficient access and connectivity make it difficult for students to continue their learning online (See Fig. 2; Chandra et al. 2020; Drane et al. 2020).

The digital inclusion report also highlights that the proportion of income required for Internet expenditure has increased faster than actual increases in income (Thomas et al. 2019). This difference has profound negative implications for those on lower or fixed incomes (Thomas et al. 2019), and this is particularly concerning in a time when an estimated 10% of the population has lost income since the pandemic onset. This report also indicates that the amount of household income spent on Internet services has also increased from 1.00% in 2014 to 1.18% in 2019 (Thomas et al. 2019) further impacting the ability of households to maintain Internet costs if there has been a loss of income due to the pandemic.

Equally important is the fact that households experiencing financial hardship may be restricted to accessing the Internet solely via mobile-only plans (rather than fixed-line). These mobile-only plans typically have lower download limits, and once these limits are exceeded, additional costs are accrued, resulting in students having limited Internet download for educational purposes. The use of such mobile-only plans is reported in 30.7% of households in the lowest income quartile (Thomas et al. 2019). The digital divide is a multifaceted concept characterised not only by differences in hardware ownership (i.e. laptops, computers) but also by differences in access and connectivity to the Internet as well as the cost, which places an additional burden on households already experiencing financial strain. Moreover, the lack of economic capital within the households puts students at higher risk of experiencing technology-related issues impacting their online learning from home.

To redress the digital divide during the COVID-19 pandemic, many countries recognised the necessity of a variety of media supporting student learning during school closure (Chandra et al. 2020; NZ Government 2020). UNESCO (2020c) has reported that countries impacted by the Ebola crisis 2014 facilitated learning environments via a range of mediums, including online avenues, as well as radio and television. Since March 2020, countries have adopted different strategies to support learning. For example, in Portugal, the government endorsed a partnership involving schools and post office services to ensure the timely delivery of hard copy teaching resources to homes (UNESCO 2020d). The New Zealand (NZ) government provided educational content via two television channels, combined with learning resources available in both hard and soft copy (NZ Government 2020). The NZ government also provided NZ$87.7 million in funding towards this endeavour (NZ Government 2020). Similarly, the Queensland government announced on 12 April 2020, in response to poor Internet connectivity, that curriculum would be taught via television, especially in rural and remote regions (Moore 2020). The programming included content to engage with parents to assist them in home-schooling their children. It is the combined interconnected processes and systems that are notable in these examples.

Clearly, rather than an exclusive Internet reliance, creative use of alternative teaching mediums is required. Such a variety of approaches may also allow for different forms of learning engagement by students. Such initiatives are also in line with another of UNESCO's recommendations: "examine the (technology) readiness and choose the most relevant tools" (UNESCO 2020b, p. 1).

In addition to differing teaching mediums, several countries have implemented loans of electronic equipment, such as laptops or tablets (Chandra et al. 2020). To assist with Internet access, pre-paid wireless Internet was also supplied in some locations (Chang and Yano 2020). The United Arab Emirates, in an attempt to assist the practical application of online teaching, opened a technical support hotline for teachers and students devised to offer free support for individuals facing difficulties with technology (UNESCO 2020d). In Italy, family members in isolation have been offered online courses aimed at relationship management (UNESCO 2020d). These practices reflect the UNESCO recommendations of "ensure inclusion of distance learning programs" and "provide support to teachers and parents on the use of digital tools" (UNESCO 2020b, p. 1).

## Technology use

Technology is rapidly evolving and therefore requires continual learning and skill development. Australian students have been varyingly exposed to technology integration. A number of misconceptions exist around the extent of technology competencies of students more broadly. While young people are often assumed to be digitally *savvy*, their technology use at home is typically for personal use and not for learning purposes (Margaryan et al. 2011; Wang et al. 2014). As technological skills vary among young people, many may not meet the required level of proficiency for learning online. Notwithstanding the discourse around the expertise of the *net generation* (Tapscott 1998) or young *digital natives* (Prensky 2001a), who have grown up with technology enmeshed within their daily lives, researchers (Bennett et al. 2008; Margaryan et al. 2011) have argued that this notion of a digital native is not commonplace. Instead, young people employ technology in ways impacted by a range of resources or capacities, including their financial, social and cultural capacity to meet their needs (Bennett et al. 2008).

**Fig. 3:**
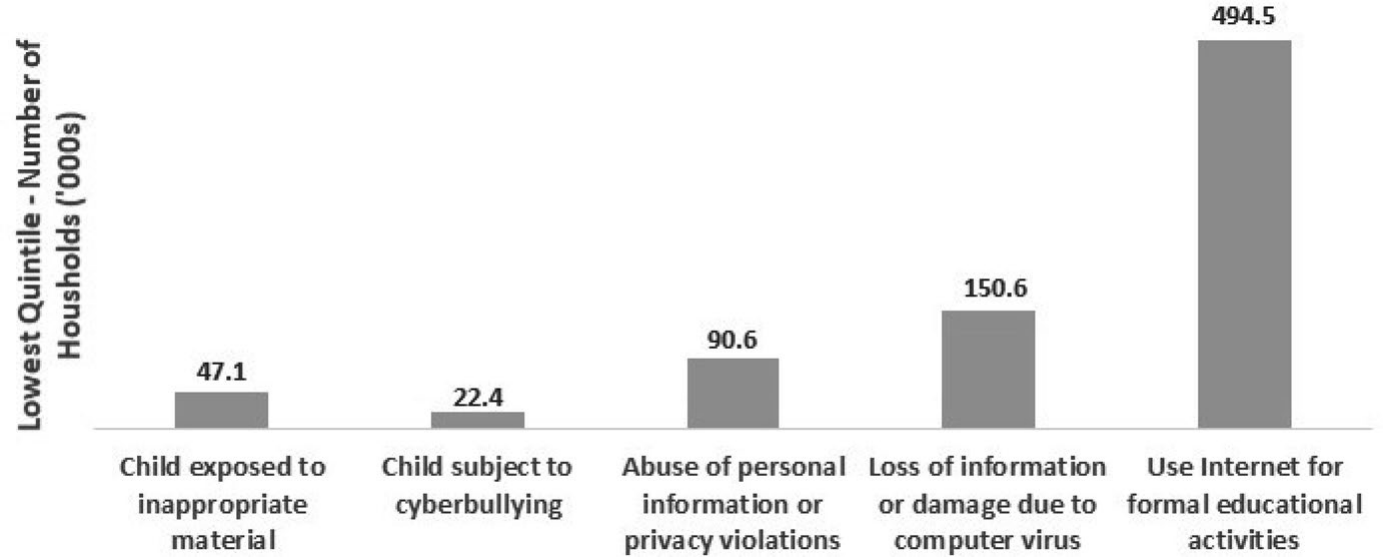
Technology Use for the Lowest Quintile of household income (ABS 2018)

Despite the conjecture that young people know how to use technology, many school-age learners may not have high levels of self-confidence to use a digital platform for learning or equally may not have acquired the necessary skills to use technology in critical ways (Thompson 2013; Wang et al. 2014; Waycott et al. 2010). Indeed, those students who can selectively access and assess technology content, that is to use technology critically, are also more likely to be students from more materially resourced backgrounds (Perotta 2013; Warschauer and Matuchniak 2010). Further, with the increased use of technology for online learning during the pandemic, there may be greater exposure to inappropriate material as well as an increased risk of cyberbullying (See Fig. 3; Drane et al. 2020). For example, 47,100 families in the lower quintile for household income have reported that their children have been previously exposed to inappropriate online material (ABS 2018; See Fig. 3; Drane et al. 2020).

A survey conducted with principals/leaders after a three-week school closure reported that flexibility in both application and structure of content varied according to different circumstances. Participants recommended avoiding a *one-stop-shop* approach to teaching content but rather recognised that bespoke strategies may be necessary to combine modalities and also reflect school priorities (Burke and Dempsey 2020). For example, in this period of the pandemic, explicitly articulating learning goals and objectives in teaching remotely is imperative for schools—for example, is the intention to teach new material or simply revise existing content? Similarly, alternative approaches to providing learning experiences should be considered as these may often be the most appropriate for those households with limited connectivity, for example, instead of using a computer, a smartphone can be used to read an email, or an instructional task can be adapted through the repurposing of resources commonly found in the home.

Several teachers and principals identified that national guidelines are required that clearly explain the expectations of schools and families over the time of closure (Burke and Dempsey 2020). These centrally developed guidelines (government or peak teaching body) must aim to address any underlying fears that schools or teachers are somehow *failing* their students during this crisis (Burke and Dempsey 2020). These approaches are reflected in UNESCO recommendations: "blend appropriate approaches and limit the number of applications and platforms and; develop distance learning rules and monitor students' learning process" (UNESCO 2020b, p. 1).

## Conclusion

Globally, we have entered a highly complex and evolving time in the provision of quality teaching and learning. As governments directed students to stay at home, schools were required to change their practices to cater for students that were now learning solely online. A range of strategies had to be quickly implemented by schools to ensure all students were safe and supported in their studies (DESE 2020). Although schools have been faced with a level of disruption not seen in generations, unlike the past, many (but not all) students have had access to technology to continue their education online. The pandemic has shown us that online education is possible. However, for students who are unable to access, or sustain the necessary engagement in online learning, the support of other learning options is essential to ensure equity for all students. Until a vaccine for COVID-19 is found, disruptions to systems that support development and well-being will remain, including uncertainties within our education system. If future disruption calls for mass school closures, then we must learn from the impact of this initial phase of COVID-19 and have systems in place to operate effectively despite being in crisis. Failure to learn from this pandemic risks exacerbating existing educational inequities and subjecting students in Australia, particularly those in vulnerable settings, to an increased risk of adverse social, emotional and behavioural outcomes. Indeed, in Australia, businesses, governments, communities, and parents have realised the intrinsic value of childcare and schools to the operation of all facets of society.

In response to mass school closures, UNESCO provided a number of recommendations to limit disruptions to education (UNESCO 2020b), and there was global evidence that countries were adopting some of these recommendations. These recommendations underpinned approaches to mass school closures that underline inclusivity, appropriate use of technology with varying modalities, the provision of support for both teachers and families, as well as the importance of creating communities that facilitate learning. However, these are recommendations only and need to be further contextualised by place-based and local knowledges of different settings. Therefore, future directions must plan for the education systems, schools, teachers, parents, and students to be prepared at multiple levels for collaborative disaster response. This must include an alternative to being physically present on the school campus, and policymakers and practitioners must ensure equity in the provision of education for all students.

Young people require a sense of stability amid rapid change to help them process, adjust, and develop new strategies for coping with emerging and fluid contexts. Attending school provides such a level of stability for many children. As we gradually move forward out of the pandemic, there is a clear need to nurture our future generations to build capacity for the disasters that will likely come again, but which we cannot anticipate. One approach that could commence immediately is governments and policymakers enlisting the talents and advocacy of youth to enable young people to set their own vision for disaster preparedness. This will be a first step to building resilience and adaptability skills to equip young people to be prepared for future crises. Proactive and multifaceted responses can best address the educational needs of our diverse student populations and also avoid widening existing educational disparities. As governments plan and prepare for future disaster responses, they must re-examine resource allocations to schools to ensure all students have equality of access to resources especially related to technology. In the short term, however, it may be prudent for schools at the local level to both recognise and tap into their communities' capacities, forming partnerships to seek solutions to issues that unfairly impact on the more vulnerable members of society.

## Footnotes

1 This research output is an extension of the original commissioned report: https://www.dese.gov.au/covid-19/schools.
